# Virology, Pathology, and Clinical Manifestations of West Nile Virus Disease

**DOI:** 10.3201/eid1108.050289b

**Published:** 2005-08

**Authors:** Edward B. Hayes, James J. Sejvar, Sherif R. Zaki, Robert S. Lanciotti, Amy V. Bode, Grant L. Campbell

**Affiliations:** * Centers for Disease Control and Prevention, Fort Collins, Colorado, USA;; † Centers for Disease Control and Prevention, Atlanta Georgia, USA

**Keywords:** West Nile virus, encephalitis, prevention, zoonosis, ecology, mosquito control, blood transfusion, organ tranplantation, pediatrics, intrauterine

## Abstract

West Nile virus (WNV) causes epidemics of febrile illness, meningitis, encephalitis, and flaccid paralysis. Since it was first detected in New York City in 1999, and through 2004, 16,000 WNV disease cases have been reported in the United States. Over the past 5 years, research on WNV disease has expanded rapidly. This review highlights new information regarding the virology, clinical manifestations, and pathology of WNV disease, which will provide a new platform for further research into diagnosis, treatment, and possible prevention of WNV through vaccination.

## Abstract 2

Virologic characteristics of WNV likely interact with host factors in the pathogenesis of fever, meningitis, encephalitis, and flaccid paralysis.

The impressive spread of West Nile virus (WNV) in the Western Hemisphere after its detection in 1999 during an outbreak of encephalitis in New York City has caused >16,000 human disease cases and >660 deaths in North America. Research on the signs, symptoms, and pathogenesis of WNV disease has greatly intensified in the past 5 years. The number of recognized cases of flaccid paralysis due to WNV infection has increased substantially, and research into prognosis and possible therapy has expanded. Genetic variation of the virus has been further characterized and continues to be explored. The pathology and pathogenesis of WNV disease have been described more completely than ever before. Several strategies are being pursued to develop effective vaccines to prevent WNV disease. This article highlights new information about the virology, clinical manifestations, laboratory diagnosis, pathology, and prognosis of WNV illness in humans. The expanded knowledge about WNV disease provides a new platform for future development of diagnostic tests, therapy, and vaccine development.

## Characteristics of West Nile Virus

WNV is an arbovirus in the family *Flaviridae*. Its spherical, enveloped capsid has a diameter of ≈50 nm and contains single-stranded RNA that encodes the capsid (C), envelope (E), and premembrane (prM) proteins, as well as 7 nonstructural proteins that likely contribute to viral replication. The virus has 2 genetic lineages: lineage 1 strains are found in North America, Europe, Africa, Asia, and Australia; lineage 2 strains have been isolated only in sub-Saharan Africa and Madagascar. Lineage 1 strains have been further divided into 4 clades: Kunjin, Indian, A, and B (which includes an Indian isolate) (1). The isolates in clade B, which includes strains from the United States, are all virulent in mice; lineage 2 and other clades in lineage 1 comprise both virulent and attenuated strains (1). Differences in pathogenicity may be related to nucleotides that code for specific regions in the prM, E, or nonstructural proteins of the virus (1,2).

WNV strains from the United States are closely related to strains from Israel, with 99.7% homology in nucleotide sequences, indicating that the strains in the United States almost certainly originated from the Middle East (3). The strain isolated in New York in 1999 is more virulent in American crows (*Corvus brachyrynchos*) than strains from Kenya and Australia (Kunjin virus, a subtype of WNV), and both the New York strain and the Kenyan strain experimentally killed house sparrows whereas the Australian strain did not (4).

Two genetic variants of the North American WNV strain were isolated in Texas in 2002; the major variant differed from the New York 1999 isolate by 0.18% of nucleotides, and the minor variant by 0.35% (1). The 2 variants differed from each other by 0.5% of nucleotides, and their neuroinvasiveness in mice was similar to that of the New York 1999 isolate. In 2003, attenuated WNV strains were found in birds in Texas and Mexico, providing the first evidence of phenotypic variation of WNV strains in the Western Hemisphere (2,5). The reduced neuroinvasiveness and smaller plaque size of the Texas strains may be due to mutations in nonstructural proteins that result in lower levels of viremia; the attenuated strain from Mexico had a mutation in the E protein (2,5).

### Pathogenesis

WNV is thought to replicate at the site of inoculation and then spread to lymph nodes and the bloodstream (6). Viral penetration of the central nervous system appears to follow stimulation of toll-like receptors and increased levels of tumor necrosis factor-α, which increases permeability of the blood-brain barrier (7). WNV directly infects neurons, particularly in deep nuclei and gray matter of the brain, brainstem, and spinal cord (8–10). Collateral destruction of bystander nerve cells may contribute to paralysis (11). Immune-mediated tissue damage may also contribute to pathologic changes in some cases (12). Genetic susceptibility for severe disease in mice has been postulated to involve a deficiency in production of 2´–5´-oligoadenylate synthetase, but this genetic susceptibility has not been elucidated in humans (10). Although most nonfatal WNV infections appear to be cleared by the host immune response, the virus may persist in some vertebrate hosts (10,13).

### Clinical Manifestations

The clinical spectrum of symptomatic WNV infection in humans has been further defined during the North American epidemics. About 80% of human infections are apparently asymptomatic (14). Of those persons in whom symptoms develop, most have self-limited West Nile fever (WNF), characterized by the acute onset of fever, headache, fatigue, malaise, muscle pain, and weakness; gastrointestinal symptoms and a transient macular rash on the trunk and extremities are sometimes reported (15,16). A recent follow-up study of WNF patients who sought medical attention found that difficulty concentrating and neck pain or stiffness were also prominent symptoms, and that fatigue and muscle weakness frequently lasted for ≈1 month after onset (16). Of the 98 patients interviewed, 31% were hospitalized, 79% missed school or work because of their illness, and the median time before patients felt fully recovered was 60 days. These patients probably represent the most severe WNF, but even without neurologic manifestations, WNV infection clearly can cause a notable public health problem, Additional nonneurologic clinical manifestations that may rarely occur during WNV infection include hepatitis, pancreatitis, myocarditis, rhabdomyolysis, orchitis, and ocular manifestations (17–24). Chorioretinitis may be more common than previously thought; a study in Tunisia found that 69% of 29 patients hospitalized with WNV disease had chorioretinitis (24). Cardiac dysrhythmias have been observed in some North American patients (Centers for Disease Control and Prevention [CDC], unpub. data) (22).

Neuroinvasive disease develops in <1% of WNV-infected persons, for example, in such forms as meningitis, encephalitis, or paralysis (the proportion of reported cases that are neuroinvasive disease is higher because neuroinvasive disease is more likely to be reported than WNF or asymptomatic infections) (14). The risk for encephalitis increases with age and is higher among organ transplant recipients (25,26). Whether other immunocompromised patients are at higher risk remains unclear, but severe WNV disease has been described in persons with malignancies (9). Whether diabetes, hypertension, and cerebrovascular disease are risk factors also remains uncertain (27). The clinical severity of WNV encephalitis ranges from mild disorientation to coma and death (28,29). Many patients with WNV encephalitis have movement disorders, including severe tremors and parkinsonism (28,29).

In ≈13% of patients with neuroinvasive WNV disease, WNV infection of spinal motor neurons (anterior horn cells) causes acute, asymmetric flaccid paralysis similar to that seen with poliomyelitis (CDC, unpub. data) (18,30,31). Infection of the brainstem and high cervical spinal cord may cause diaphragmatic and intercostal muscle paralysis with resulting respiratory failure and sometimes death. A separate syndrome consistent with acute inflammatory demyelinating polyradiculoneuropathy (Guillain-Barré syndrome) has been infrequently reported (32).

### Pathologic Changes

Histologic findings of WNV encephalitis include perivascular inflammation, microglial nodules, variable necrosis, and loss of neurons (Figure panels A, B) (8,9). The deep gray nuclei, brainstem, and spinal cord appear to be most affected (8,9). Patients with flaccid paralysis have perivascular lymphocytic infiltration in the spinal cord, microglial nodules, and loss of anterior horn cells (9). Spinal cord inflammation was seen in 17 of 23 people who died with WNV neuroinvasive disease; inflammation was more prominent in the anterior horns than in the posterior horns of 9 patients (9). Endoneural mononuclear inflammation of cranial nerve roots and spinal nerves can be found in a small percentage of persons. Foci of demyelination, gliosis, and occasional perivascular infiltrates may be found in persons with prolonged clinical courses.

**Figure:**
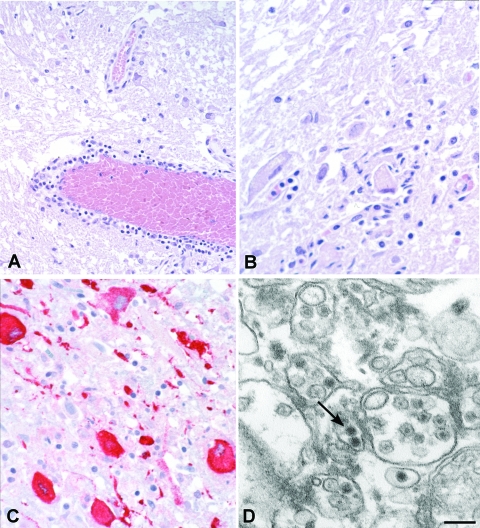
Histopathologic features of West Nile virus (WNV) in human tissues. Panels A and B show inflammation, microglial nodules, and variable necrosis that occur during WNV encephalitis; panel C shows WNV antigen (red) in neurons and neuronal processes using an immunohistochemical stain; panel D is an electron micrograph of WNV in the endoplasmic reticulum of a nerve cell (arrow). Bar = 100 nm.

Before 2001, attempts to isolate WNV from postmortem tissues in the United States had been unsuccessful. Recently, the virus has been isolated postmortem from 2 immunosuppressed patients with apparently high viral loads (33). Immunohistochemical (IHC) staining is more sensitive than viral culture, showing WNV antigens in ≈50% of fatal WNV neuroinvasive disease cases; IHC staining is particularly useful in patients who died during the first week of illness when viral antigen concentrations in central nervous system (CNS) tissues are high (9). Viral antigens are usually found within neurons and neuronal processes, predominantly in the brain stem and anterior horns (Figure, panel C). In general, antigens are focal and sparse, except in immunosuppressed patients in whom they can be seen extensively throughout the CNS (9). Visualization of WNV particles by electron microscopy is rare. When found, they are seen within endoplasmic reticulum of neurons (Figure, panel D).

### Diagnostic Tests

Routine clinical laboratory studies do not distinguish WNV infection from many other viral infections. Patients with neuroinvasive disease generally have lymphocytic pleocytosis in the cerebrospinal fluid (CSF), but neutrophils may predominate early in the course of illness (28,31). Results of brain magnetic resonance imaging are frequently normal, but signal abnormalities may be seen in the basal ganglia, thalamus, and brain stem of patients with encephalitis, and in the anterior spinal cord in patients with poliomyelitislike syndrome (18,29,31). Clinical features and electrodiagnostic tests can help differentiate poliomyelitislike syndrome from Guillain-Barré syndrome by localizing damage primarily to motor axons, anterior horn cells, or both, with relative sparing of sensory nerves in the former, as opposed to localizing the damage to peripheral myelin or muscle in the latter (18,31,32,34).

Detection of WNV-specific immunoglobulin (Ig) M in serum or CSF provides strong evidence of recent WNV infection. In most patients, IgM antibody against WNV is usually detectable by 8 days after illness onset; however, in patients with WNV neuroinvasive disease, specific IgM is almost always detectable in serum and CSF by the time CNS symptoms begin (35). Among asymptomatic WNV-viremic blood donors who were seronegative at the time of donation, IgM appeared ≈9 days postdonation, and IgG appeared ≈4 days later (M. Busch, pers. comm.). IgM is detectable in serum of ≈36% of patients who have survived WNV encephalitis at 12 months postonset and ≈20% at 16 months postonset; IgM is also detectable in CSF of other patients up to 199 days postonset (36,37). Consequently, detectable IgM may occasionally reflect past rather than recent infection.

Recently developed microsphere immunoassays for WNV antibody appear to be more accurate and efficient than current enzyme immunoassays (EIAs) (J. Johnson, pers. comm.) (38). As with standard EIA, related flaviviral infection may elicit cross-reactive test results. A microsphere assay with nonstructural viral antigens appears to discriminate between primary flaviviral infections that elicit cross-reactive antibody to the E glycoprotein (38).

A ≥4-fold change in virus-specific neutralizing antibody titer (detected by plaque-reduction neutralization test [PRNT]) between 2 serum specimens collected 2–3 weeks apart usually confirms acute WNV infection. Samples with WNV-specific antibody will usually have neutralizing antibody titers to WNV that are >4-fold higher than titers to other epidemiologically relevant flaviviruses included in the assay. However, PRNT may not discriminate between WNV infection and other flaviviral infections in patients with previous flavivirus exposure, because the neutralizing antibody in such cases may broadly cross-react to several related flaviviruses.

WNV infection can also be diagnosed by detecting virus in CSF, serum, or tissues by isolation or nucleic acid amplification tests (NATs). WNV is best isolated in cell culture or suckling mice and identified by indirect immunofluorescence assay with specific monoclonal antibodies or by reverse transcriptase–polymerase chain reaction (RT-PCR). However, WNV is rarely isolated from the blood of patients with neuroinvasive WNV disease because viremia levels are typically low or absent by the time neurologic symptoms develop. Real-time RT-PCR and nucleic acid sequence-based amplification are the most sensitive NATs, able to detect ≥50 viral RNA copies per mL (≈0.1 PFU/mL), which is ≈1,000-fold more sensitive than culture (39). WNV can be detected in serum by NAT if the specimen is obtained early in infection and is readily detected by NAT, isolation, or IHC staining in brain tissue from persons with fatal cases. The sensitivity of RT-PCR among 28 patients with serologically confirmed neuroinvasive WNV disease was 57% in CSF and 14% in serum (40).

The diagnosis of WNV encephalitis can be supported histopathologically, and there is no pathognomonic lesion. Differential diagnoses include arboviral and other viral encephalitides, rickettsial infections, and various noninfectious diseases. When serum samples and frozen tissues are not available, IHC testing of formalin-fixed tissues with specific monoclonal and polyclonal antibodies is particularly useful.

### Prognosis

The clinical course of WNF ranges from a mild febrile illness of several days' duration to debilitating fatigue, aching, and weakness that may last for weeks or months (16,29,41). Although cases of meningitis without alteration of the patient's mental status or other focal neurologic features have a favorable prognosis, persistent headaches and fatigue may be reported (29). Patients with WNV encephalitis or focal neurologic manifestations often have persistent neurologic deficits for months or years (28,29). Of 35 patients hospitalized with WNV disease in New York, only 13 (37%) reported full recovery in physical, cognitive, and functional abilities 12 months after illness onset (41). Many patients with WNV-associated poliomyelitislike syndrome do not recover, but some improvement in limb strength may occur over time (42,43). The overall case-fatality rate for neuroinvasive WNV disease is ≈9% (26).

### Clinical Management

Management of severe WNV illness remains supportive. Patients with severe meningeal symptoms often require pain control for headaches and antiemetic therapy and rehydration for associated nausea and vomiting. Patients with severe encephalitis should be observed for development of elevated intracranial pressure and seizures, and patients with encephalitis or paralysis must be monitored for inability to protect the airway. Acute neuromuscular respiratory failure may develop rapidly, particularly in patients with prominent bulbar signs; prolonged ventilatory support may be required (22,30,34).

Ribavirin, interferon-α, WNV-specific immunoglobulin, and antisense gene–targeted compounds have all been considered as specific treatments for WNV disease, but no rigorously conducted clinical trials have been completed. Nonspecific immunoglobulin and plasmapheresis should be considered for patients with Guillain-Barré syndrome but are not indicated for patients with paralysis due to damage of anterior horn cells (30).

### Vaccine Development

Two vaccines are available for vaccinating equines: an inactivated WNV vaccine and a recombinant vaccine that uses canarypox virus to express WNV antigens (44,45). An inactivated vaccine is also being studied for use in humans (46). A chimeric live virus vaccine incorporating the genetic sequences for E and prM antigens into a 17-D yellow fever virus backbone has been shown to be efficacious in hamsters and is undergoing initial clinical trials in humans (46). Another chimeric vaccine incorporating WNV genetic sequences into a backbone of attenuated serotype-4 dengue virus–induced protective immunity in monkeys (44). A DNA vaccine that elicits expression of WNV E and prM antigens has been used in mice, horses, and birds (44). Vaccination of crows with Kunjin virus, a subtype of WNV, protected against WNV, and a DNA vector, which elicited expression of attenuated Kunjin virus, provided protective immunity against WNV in mice (46).

### Future Directions

Since the 1990s, WNV has gained notoriety as a cause of severe neuroinvasive disease in humans. As WNV isolates and genetic sequences accumulate over an increasing geographic and clinical range, the virus shows signs of genetic modifications that likely interact with host factors in causing different patterns of neuroinvasiveness and neurovirulence. Several areas warrant research focus over the next few years. More efficient diagnostic assays will help with both clinical diagnosis and disease surveillance. Improved knowledge about the pathogenesis and natural history of WNV disease is crucial to developing effective treatment, and promising therapies need to be carefully evaluated in controlled clinical trials. Given the focal distribution of WNV outbreaks, and the uncertain distribution of future cases of WNV disease, prospective clinical studies need to be designed with the flexibility to gather information from widely dispersed and changing locations. The development of a safe and effective vaccine for humans is a clear priority for prevention, and the public health strategies and recommendations for vaccination deserve careful thought. Given the relatively low incidence of WNV neuroinvasive disease and the focal occurrence of WNV epidemics thus far, vaccination will likely require targeting to higher risk groups to approach the cost-effectiveness of many recommended public health prevention strategies.
